# Study protocol: evaluation of a parenting and stress management programme: a randomised controlled trial of Triple P Discussion Groups and Stress Control

**DOI:** 10.1186/1471-2458-13-888

**Published:** 2013-09-25

**Authors:** Melanie L Palmer, Marion Henderson, Matthew R Sanders, Louise J Keown, Jim White

**Affiliations:** 1 Medical Research Council/Chief Scientist Office Social and Public Health Sciences Unit, University of Glasgow, 4 Lilybank Gardens, Glasgow G12 8RZ, United Kingdom; 2 School of Learning, Development, and Professional Practice, Faculty of Education, The University of Auckland, Private Bag 92601 Symonds St, Auckland 1150, New Zealand; 3 STEPS Primary Care Mental Health Team, National Health Service Greater Glasgow and Clyde, 60 Florence Street, Glasgow G5 0YX, United Kingdom

## Abstract

**Background:**

Children displaying psychosocial problems are at an increased risk of negative developmental outcomes. Parenting practices are closely linked with child development and behaviour, and parenting programmes have been recommended in the treatment of child psychosocial problems. However, parental mental health also needs to be addressed when delivering parenting programmes as it is linked with parenting practices, child outcomes, and treatment outcomes of parenting programmes. This paper describes the protocol of a study examining the effects of a combined intervention of a parenting programme and a cognitive behavioural intervention for mental health problems.

**Methods/design:**

The effects of a combined intervention of Triple P Discussion Groups and Stress Control will be examined using a randomised controlled trial design. Parents with a child aged 3–8 years will be recruited to take part in the study. After obtaining informed consent and pre-intervention measures, participants will be randomly assigned to either an intervention or a waitlist condition. The two primary outcomes for this study are change in dysfunctional/ineffective parenting practices and change in symptoms of depression, anxiety, and stress. Secondary outcomes are child behaviour problems, parenting experiences, parental self-efficacy, family relationships, and positive parental mental health. Demographic information, participant satisfaction with the intervention, and treatment fidelity data will also be collected. Data will be collected at pre-intervention, mid-intervention, post-intervention, and 3-month follow-up.

**Discussion:**

The aim of this paper is to describe the study protocol of a randomised controlled trial evaluating the effects of a combined intervention of Triple P Discussion Groups and Stress Control in comparison to a waitlist condition. This study is important because it will provide evidence about the effects of this combined intervention for parents with 3–8 year old children. The results of the study could be used to inform policy about parenting support and support for parents with mental health problems.

**Trial registration:**

ClinicalTrial.gov: NCT01777724, UTN: U1111-1137-1053.

## Background

Young children displaying psychosocial problems (problems with behaviour, emotions, and relationships) are a significant public health concern due to the associated costs to the individual and society [1,2]. Of great concern is the large proportion of young children who display psychosocial problems. Prevalence rates from the Growing Up in Scotland (GUS) study found between 10-27% of children at primary school entry display emotional or behavioural problems considered to be outside the normal range, with conduct problems being the most prevalent difficulty [3]. As parenting practices are inextricably linked with child development and behaviour, parenting programmes have been recommended as the preferred treatment for young children displaying psychosocial problems [4].

Parents’ mental health is also linked with both parenting practices and child outcomes. Reports based on the GUS study stated that at any one time, approximately 12-16% of mothers of young children experienced mental health problems and that children with mothers with mental health problems were more likely to have negative behavioural, emotional, and peer outcomes than children whose mothers were without mental health problems [5]. It may be that parents’ inability to cope with mental health problems may limit their capacity to carry out effective parenting strategies and be positive in their interactions with their children [6].

Caring for a child with psychosocial problems can also be a depressing and stressful experience. Research has found that parents with a child who displays very difficult behaviour are more likely to report having a stressful or depressing parenting experience [7]. Other research has examined the relationship between parenting stress and child behaviour problems and found that parenting stress and child behaviour problems are both antecedents and consequences of one another, and therefore, have a mutually escalating effect over time [8], and that child behaviour problems predict a large amount of variance in parenting stress [9]. Furthermore, in efforts to manage difficult child behaviours parents use more coercive parenting practices [10]. It could be that less optimal parenting practices are an indirect outcome of parents’ limited capacity to cope with stress, including the stress related to dealing with their child’s psychosocial problems [6].

Previous researchers have suggested that parental mental health is particularly important to take into account when delivering parenting programmes as parents are responsible for implementing parenting strategies and modifying children’s behaviour [11]. There is also evidence that poorer parent mental health or parenting stress is associated with poorer treatment outcomes for children following parenting programmes [12-14]. This highlights the importance of addressing parents’ mental health alongside parenting practices when treating children displaying psychosocial problems.

One way to improve both parenting practices and parental mental health and maximise treatment outcomes of parenting programmes is to deliver parenting programmes in combination with a cognitive behavioural intervention aimed to improve mental health problems. There is some research that has examined the effects of combined interventions that target both parenting and mental health problems and reported reductions in dysfunctional/ineffective parenting practices, parenting stress, child problem behaviours, and improvements in parents’ mental wellbeing e.g., [15,16]. However, the interventions delivered in these studies are high-intensity interventions and take up a lot of individual practitioner time.

In contrast to high-intensity interventions, low-intensity interventions refer to programmes that require a low usage of practitioner time or usage of time in a cost-effective way, such as group based programmes [17]. Low-intensity interventions aim to increase access to evidence-based practice to enhance health and wellbeing on a population-wide basis [17]. Some low-intensity interventions are entirely untargeted, whereas others are targeted towards a particular group of individuals. To our knowledge, there is no research examining whether a combination of a low-intensity parenting programme and a low-intensity cognitive behavioural intervention for mental health problems would be effective in improving parenting practices, parents’ mental wellbeing, and children’s psychosocial problems. Thus the aim of the proposed study is to determine the effects of a low-intensity parenting programme (Triple P Discussion Groups, described below in the intervention section) and a low-intensity cognitive behavioural intervention for mental health problems (Stress Control, described below).

### Objective

This paper aims to describe the study protocol of a randomised controlled trial (RCT) of a combined intervention of Triple P Discussion Groups and Stress Control for parents with 3–8 year old children.

## Methods/design

### Design

**Figure 1:**
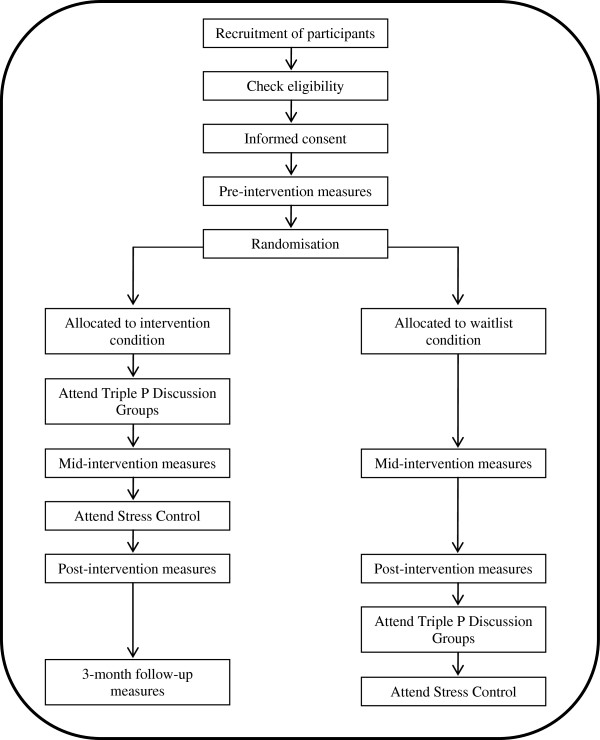
Study design.

The study is designed as a feasibility RCT (see Figure 1). First, informed consent will be obtained from all participants, followed by pre-intervention measures being administered. After pre-intervention measures are completed, participants will be randomly assigned to either an intervention or a waitlist condition. The intervention condition receives the combined intervention of Triple P Discussion Groups and Stress Control immediately after pre-intervention measures, whereas, the waitlist condition receives the intervention approximately 12 weeks later. To evaluate the effects of the intervention in comparison to a waitlist, participants in the intervention condition will complete questionnaire measures at mid-intervention and post-intervention. Participants in the waitlist condition will complete the same measures at the equivalent times. Participants allocated to the intervention condition will also complete questionnaire measures at 3-month follow-up. The National Health Service (NHS) West of Scotland Research Ethics Committee (REC ref 12/WS/0242) and the NHS Research and Development Management Office (R&D ref GN12FS463) have approved the study protocol and documentation.

### Participants

Eligible participants will be parents/carers with a 3–8 year old child in Glasgow, Scotland. We aim to recruit 160 parents to participate in the study.

### Recruitment of study population

Advertising materials (a brochure, a poster, a flyer, and a blurb about the study) have been developed to inform potential participants of the study and encourage them to self-refer to participate. The advertisements will be disseminated to the local community in several ways: 1) the Stress Control website and members of the NHS Greater Glasgow and Clyde (GGC) STEPS Primary Care Mental Health Team (http://www.glasgowsteps.com), 2) GPs and health clinics in local community, 3) community health teams in the South Glasgow area (multi-disciplinary teams of social care, nursing, and health staff who provide support and advice to families with young children: http://www.chps.org.uk/glasgow), 4) local primary schools, nurseries, early years centres, and playgroups, 5) libraries, cafes, and shops in the local community, and 6) social media. The advertisements encouraged parents with 3–8 year old children who were *‘juggling a lot as well as being a parent’* and were interested in attending 8-week group programme aimed at helping parents *‘learn to relax, de-stress, and achieve Healthy And Positive Parenting for You’* to self-refer to take part. Upon contact, the researchers will inform parents interested in taking part of the study protocol and obtain informed consent. Recruitment of participants to the study will start in March 2013.

### Study inclusion and exclusion criteria

To take part in the study, participants must: 1) have a child between the ages of 3 and 8 years old (rationale: the content delivered in the Triple P Discussion Groups is relevant for parents with children between the ages of 3 and 8 years), 2) be able to attend the group sessions held at the Langside Halls in Shawlands, Glasgow, and 3) be able to read a newspaper without assistance (rationale: the written materials used in the Triple P Discussion Groups and Stress Control are not suitable for parents who cannot read a newspaper without assistance). Participants are excluded if the child has a diagnosis of a developmental or intellectual disability or other significant health impairment (rationale: the Triple P Discussion Groups are designed for children with psychosocial problems that are otherwise normally developing).

### Randomisation procedure

Randomisation occurs at the level of individual target children. A computer generated list of random numbers will be used to allocate to condition in sequence of completion of pre-intervention measures. Allocation to condition will be conducted by an individual independent of the study to ensure there is no bias in the allocation.

### Sample size

The Parenting Scale (PS) Total score and the Depression Anxiety and Stress Scales-21 (DASS-21) Total score were used to determine the sample size. For a 0.3-point difference between the two conditions at post-intervention on the PS Total score, given a standard deviation of 0.6 (an effect size of 0.5), 64 families per condition are required to achieve power of 80% at an alpha of .05. For a 5-point difference between the two conditions at post-intervention on the DASS-21 Total score, assuming a standard deviation of 10 (an effect size of 0.5), 64 families per condition are required to achieve power of 80% at an alpha of .05. Assuming expected attrition of 20%, an initial sample of 160 families is needed, thus 80 families in each condition. Table 1 displays the expected means and standard deviations for the PS Total and DASS-21 Total scores for the two conditions at pre- and post-intervention.

**Table 1:** Expected mean and standard deviations for the primary outcome measures

	**Intervention condition**				**Waitlist condition**			
	**Pre-intervention**		**Post-intervention**		**Pre-intervention**		**Post-intervention**	
	***M***	***SD***	***M***	***SD***	***M***	***SD***	***M***	***SD***
Expected PS Total score	3.0	0.6	2.7	0.6	3.0	0.6	3.0	0.6
Expected DASS-21 Total score	18	10	13	10	18	10	18	10

### Intervention

The intervention to be evaluated is a combination of Triple P Discussion Groups and Stress Control. The Triple P-Positive Parenting Program is a public health approach to preventing and treating emotional, behavioural and developmental problems displayed by children and adolescents [18] (http://www.triplep.net). This is achieved through increasing knowledge, skills, and confidence in parenting. Triple P uses a tiered system of intervention with five levels of increasing strength, ranging from information-based strategies delivered via the media (level 1) to intensive individually tailored multi-session programmes (level 5). Four meta-analyses examining the outcomes of Triple P interventions have demonstrated positive effects for child behaviour problems, parenting practices and self-efficacy, and parental wellbeing [19-22].

The Triple P Discussion Groups are a level 3, low-intensity intervention designed to provide specific advice about common child behaviour or developmental issues. The Triple P Discussion Groups are interactive in nature and are typically run with 10–20 parents. Previous research evaluating the Triple P Discussion Groups using randomised controlled trial designs has found that in comparison to a control group, participants in the intervention condition reported significantly fewer child behaviour problems, less dysfunctional/ineffective parenting practices, and greater parenting self-efficacy after attending the programme [23,24].

Two 120 minute Triple P Discussion Groups will be used in this study; the ‘Being a Positive Parent’ and the ‘Dealing with Disobedience’ Triple P Discussion Groups (see Table 2 for information about session content, duration, and delivery). All practitioners delivering the Triple P Discussion Groups will be trained in Triple P and standardised training is provided by Triple P International (for more information on training see: http://www.triplep.net).

Stress Control is a low-intensity cognitive behavioural intervention that aims to promote mental health and wellbeing on a community-wide basis [25] (http://www.glasgowsteps.com). The central focus of Stress Control is to teach cognitive behavioural therapy techniques to help individuals cope with anxiety, depression, panic, poor sleep, and/or low self-confidence. The programme is didactic in nature, consists of six 90 minute group sessions, and is typically delivered to 30–100 people [25] (see Table 2 for information about session content, duration, and delivery). Research evaluating Stress Control using controlled trial designs and pre-post uncontrolled designs has found that Stress Control is effective in reducing participants’ anxiety, depression, and distress, and improves general psychological wellbeing [25-28]. The practitioners delivering the Stress Control sessions are staff from the NHS GGC STEPS Primary Care and Mental Health Team. The STEPS Primary Care and Mental Health Team provide a range of mental health services to individuals in South East Glasgow and Stress Control is delivered as part of their regular practice. All practitioners delivering the Stress Control sessions are trained to deliver the programme. Training and supervision is provided by the programme developer, Dr Jim White, a member of the STEPS Primary Care and Mental Health Team.

**Table 2:** Overview of intervention sessions

**Session**	**Content**	**Duration**	**Delivered by**
Being a Positive Parent Triple P Discussion Group	• Introduction to principals of positive parenting	120 minutes	Trained Triple P practitioners employed by the NHS GGC
	• Taught skills to support child’s competence and development, and build a positive relationship with their child		
Dealing with Disobedience Triple P Discussion Group	• Introduction to reasons for child disobedience and parenting traps		
	• Taught skills to encourage positive child behaviour and to manage disobedience		
Stress Control Session 1: Information about stress	• Introduction to Stress Control	90 minutes	Employees of the NHS GGC, STEPS Primary Care and Mental Health Team
	• Information about common mental health problems provided		
Stress Control Session 2: Controlling your body	• Introduction to how stress affects your body		
	• Taught skills to control your body		
Stress Control Session 3: Controlling your thoughts	• Introduction to how stress affects your thoughts		
	• Taught skills to control your thoughts		
Stress Control Session 4: Controlling your actions	• Introduction to how stress affects your actions		
	• Taught skills to control your actions		
Stress Control Session 5: Controlling your panic, using your breathing to control stress, prevention skills and medication	• Introduction to panic and stress		
	• Taught skills to control panic		
	• Information on medications and antidepressants is provided		
Stress Control Session 6: Controlling your sleep, wellbeing, and controlling your future	• Introduction to how stress affects your sleep		
	• Introduction to wellbeing		
	• Skills to control your sleep and managing stress in the future		

### Intervention condition

Families allocated to the intervention condition will receive the intervention immediately after the pre-intervention measures. The intervention is delivered over an eight week period with one session per week. The Triple P Discussion Groups are delivered first followed by Stress Control. We assumed that participants would be more likely to drop out towards the end of the intervention and therefore decided that the Triple P Discussion Groups should be delivered first to ensure most participants were exposed to at least some of each intervention. The mid-intervention questionnaire will be administered at the end of the Triple P Discussion Group sessions but prior to the start the Stress Control sessions (i.e., approximately two weeks after the start of the intervention). Post-intervention measures will be administered immediately after the end of the Stress Control sessions, and a follow-up questionnaire approximately 3-months later. The duration of the study for participants in the intervention condition is from referral until the 3-month follow-up questionnaire. For these participants, the approximate length of the time in the study is 24 weeks. Participants allocated to the intervention condition are able to utilise any other service during the duration of the study. This may include services aimed to improve their mental health, their parenting, or their child’s behaviour, and participants will be asked about use of other services.

### Waitlist condition

Families allocated to the waitlist condition will be asked to wait approximately 12 weeks before they participate in the intervention. During the 12 weeks, participants in the waitlist condition will complete questionnaire measures at two time points; at the equivalent times of mid- and post-intervention questionnaires for the intervention group. After the post-intervention equivalent questionnaire has been completed, participants will be offered the intervention. The duration of the study for participants in the waitlist control condition is from referral until the end of the intervention. For these participants, the approximate length of the time in the study is 20 weeks. Like participants in the intervention condition, participants allocated to the waitlist condition are able to utilise any other service during the duration of the study and information about their use of other services will be obtained.

### Measures

A description of the measures used in the study, the time points of administration, and the rational for use is reported in Table 3. As the combined intervention of Triple P Discussion Groups and Stress Control has a dual focus on parenting practices and mental health, there are two primary outcomes of the study: change in dysfunctional/ineffective parenting practices and change in symptoms of depression, anxiety, and stress. Secondary outcome measures assess change in child behaviour, parenting experiences, parental self-efficacy, xfamily relationships, and positive parental mental health. Other measures include family demographics and participant satisfaction with the intervention. For families with two or more parents/carers, only one parent/carer will be asked to complete the outcome measures, although all parents/carers will be encouraged to participate in the intervention. If there is more than one child in the family in the target age range who meets the eligibility criteria, the participant will be asked to choose a target child (the one who is the most cause for concern) to complete the measures about.

Treatment fidelity of the Triple P Discussion Groups and Stress Control sessions will also be measured (see Table 3). The practitioners delivering the sessions will complete a session content checklist after the end of each session and the percentage of content covered will be calculated. In addition, all group sessions will be audio-recorded and approximately 30% of the recorded sessions will be randomly checked by an independent observer. Adherence to the intervention content will be recorded using the same session checklist. Agreement between the practitioner-com-pleted session checklist and the session checklist completed by the independent observer will be examined to determine the extent of inter-observer agreement on treatment fidelity.

**Table 3:** Measures and data collection points

**Construct**	**Measure**	**Administration time point**				**Rationale for use**
		**Pre-**	**Mid-**	**Post-**	**follow-up**	
		**intervention**	**intervention**	**intervention**	**follow-up**	
*Primary outcome measures*						
Dysfunctional/ineffective parenting practices	The Parenting Scale (PS) [29]	✓	✓	✓	✓	To measure the effects of the combined intervention on dysfunctional/ineffective parenting practices
Parent mental health	Depression Anxiety Stress Scales-21 (DASS-21) [30]	✓	✓	✓	✓	To measure the effects of the combined intervention on symptoms of depression, anxiety, and stress
*Secondary outcome measures*						
Disruptive child behaviour	Eyberg Child Behavior Inventory-Intensity Scale (ECBI-I) [31]	✓		✓	✓	To measure the effects of the combined intervention on children’s behaviour
Parenting experiences	Parenting Experience Survey (PES) [32]	✓		✓	✓	To measure the effects of the combined intervention on parenting experiences
Parental self-efficacy	Child Adjustment and Parent Efficacy Scale-Parent Efficacy subscale (CAPES-PE) (Morawska A, Sanders MR, Haslam D, Filus A, Fletcher R: Child Adjustment and Parent Efficacy Scale (CAPES): development and initial validation of a parent report measure, submitted)	✓		✓	✓	To measure the effects of the combined intervention on parental self-efficacy
Family relationships	Parenting and Family Adjustment Scale-Family Relationships subscale (PAFAS-FR) [33]	✓		✓	✓	To measure the effects of the combined intervention on family relationships
Positive parent mental health	Short Warwick-Edinburgh Mental Well-being Scale (SWEMWBS) [34]	✓		✓	✓	To measure the effects of the combined intervention on parental positive mental health
*Other measures*						
Family demographics	Family Background Questionnaire	✓				To describe the demographic information of the participating families
Participant satisfaction with the Triple P Discussion Groups	Triple P Client Satisfaction Questionnaire (CSQ) [35]			✓		To measure the acceptability and satisfaction with the Triple P Discussion Group sessions
Participant satisfaction with Stress Control	Stress Control Client Satisfaction Questionnaire			✓		To measure the acceptability and satisfaction with the Stress Control sessions
		**Procedure**				
Treatment fidelity of Triple P Discussion Groups	Session content checklist [36]	Completed by the practitioner/s at the end of each Triple P Discussion Group				To determine treatment fidelity and integrity of the Triple P Discussion Group sessions
Inter-observer agreement of treatment fidelity of Triple P Discussion Groups	Session content checklist [36]	All Triple P Discussion Group sessions will be audio-recorded and approximately 30% of the recorded sessions will be randomly checked by an independent observer. Adherence to the intervention content will be recorded and inter-observer agreement calculated				To determine inter-observer agreement on the session content checklist designed to determine treatment fidelity of the Triple P Discussion Group sessions
Treatment fidelity of Stress Control	Session content checklist - developed for this study	Completed by the practitioner/s at the end of each Stress Control session				To determine treatment fidelity and integrity of the Stress Control sessions
Inter-observer agreement of treatment fidelity of Stress Control	Session content checklist - developed for this study	All Stress Control sessions will be audio-recorded and approximately 30% of the recorded sessions will be randomly checked by an independent observer. Adherence to the intervention content will be recorded and inter-observer agreement calculated				To determine inter-observer agreement on the session content checklist designed to determine treatment fidelity of the Stress Control sessions

### Data collection procedure

Information from participants will be obtained through questionnaires. Questionnaires will be administered in a variety of ways including during home visits, via the telephone, hardcopy via post, and online, depending on each participant’s preference. Contact with participants will be made via email and phone if questionnaires are not completed and returned.

### Data analysis procedure

Descriptive statistics will be used to present participation and retention rates. Means and standard deviations will be presented for continuous outcome measures and frequencies and percentages will be presented for categorical variables. The pre-intervention characteristics of participants in each condition (intervention and waitlist) will be compared. Chi-squared tests will be used to compare any group differences for categorical variables and *t*-tests will be used for continuous variables. Missing data will be explored following data collection and depending on the amount and type of missing data, a range of methods may be utilised. It is likely that imputation techniques will be used to extrapolate missing data.

Univariate and multivariate analyses will be conducted to examine the effects of the combined intervention in comparison to the waitlist condition on changes in dysfunctional/ineffective parenting practices, parental mental health, children’s behaviour, parenting experiences, parenting self-efficacy, family relationships, and parental positive mental health. All analyses will use an intent-to-treat approach using data from all participants who completed pre-intervention measures. In addition to intent-to-treat analyses, per protocol analyses will be conducted using data from participants who attended at least one session of the intervention and who completed measures at more than one time point. SPSS will be used to conduct the analyses and estimates of effects will be calculated and reported using Cohen’s *d*. Reporting of the trial will follow the CONSORT guidelines.

### Time frame for the study

The total duration of the study will be 18–21 months. First, ethics approval and preparatory work will take approximately 6 months. Recruitment will start March 2013 will last 6 months. Follow-up will last 3 months. We anticipate it will take between 3 and 6 months to analyse the data and report the findings. The duration of participation in the study for participants will be approximately 20–24 weeks.

## Discussion

The aim of this paper was to describe the study protocol of a feasibility RCT of a combined intervention of Triple P Discussion Groups and Stress Control in comparison to a waitlist condition, for parents with 3–8 year old children. By conducting this study, we hope to add to the literature on low-intensity parenting programmes and low-intensity cognitive behavioural interventions for mental health problems and explore their combined effects for parents with young children. As this study is a feasibility trial, it is our aim that if the combined intervention is effective, a trial with a larger sample of participants will be conducted. Furthermore, the results of the study could inform future research and policies regarding parenting support and support for parents with mental health problems. For example, the model for intervention could be adopted by the NHS GGC and included as part of their routine practice.

### Strengths

A key strength of the study is the originality of the research. We are not aware of any literature that evaluates the effects of a combined intervention of Triple P Discussion Groups and Stress Control, or a combination of any low-intensity parenting programme and low-intensity cognitive behavioural intervention to manage stress, anxiety, depression, panic, poor sleep and/or low self-confidence. Another strength of the study is the use of a RCT design to examine the effects of the intervention. Random allocation to condition reduces the likelihood of systematic differences between conditions as well as selection and allocation bias.

### Limitations

There are some limitations with this study. As the study is a two arm feasibility RCT, we are unable to evaluate the effects of the combined intervention of Triple P Discussion Groups and Stress Control in comparison to the Triple P Discussion Groups as a standalone intervention and Stress Control as a standalone intervention. Therefore, we cannot determine whether the combined intervention produces effects over and above either intervention alone. Another potential limitation is that the order of the Triple P Discussion Groups and Stress Control will remain constant in this study. It may be that for parents to achieve maximum benefits from a parenting programme, mental health problems first need to be addressed. Alternatively, teaching effective parenting and child management skills first may reduce symptoms of depression, anxiety and stress and increase parents’ mental wellbeing.

## Competing interests

Matthew R Sanders is the founder and lead author of the Triple P-Positive Parenting Program and Jim White is the founder and lead author of Stress Control. Melanie L Palmer, Marion Henderson, and Louise J Keown declare no competing interests.

## Authors' contributions

All authors contributed to the design of the study protocol. MLP wrote the final manuscript, which was reviewed by all other authors. All authors read and approved the final manuscript.

## Pre-publication history

The pre-publication history for this paper can be accessed here:

http://www.biomedcentral.com/1471-2458/13/888/prepub
