# Erratum to: Study protocol: evaluation of a parenting and stress management programme: a randomised controlled trial of Triple P Discussion Groups and Stress Control

**DOI:** 10.1186/s12889-015-1507-x

**Published:** 2015-03-18

**Authors:** Melanie L Palmer, Marion Henderson, Matthew R Sanders, Louise J Keown, Jim White

**Affiliations:** Medical Research Council/Chief Scientist Office Social and Public Health Sciences Unit, University of Glasgow, 4 Lilybank Gardens, Glasgow, G12 8RZ UK; School of Learning, Development, and Professional Practice, Faculty of Education, The University of Auckland, Private Bag 92601 Symonds St, Auckland, 1150 New Zealand; STEPS Primary Care Mental Health Team, National Health Service Greater Glasgow and Clyde, 60 Florence Street, Glasgow, G5 0YX UK

## Erratum

To ensure transparency the authors wish to include the following additional disclosure statement that was not included in the final publication [1]. The Triple P-Positive Parenting Program is owned by The University of Queensland. The University, through its technology transfer company Uniquest Pty Ltd., has licensed Triple P International Pty Ltd to disseminate the program worldwide. Royalties stemming from this dissemination work are distributed to the Faculty of Health and Behavioural Sciences, the School of Psychology, Parenting and Family Support Centre, and contributory authors in accordance with the University’s intellectual property policy. No author has any share or ownership in Triple P International.

Matthew Sanders is the founder and an author on various Triple P programs and a consultant to Triple P International. Jim White is the founder and lead author of Stress Control. Melanie L Palmer, Marion Henderson, and Louise J Keown declare no competing interests.

## References

[1] Palmer ML, Henderson M, Sanders MR, Keown LJ, White J (2013). Study protocol: evaluation of a parenting and stress management programme: a randomised controlled trial of Triple P Discussion Groups and Stress Control. BMC Public Health.

